# High rates of loss to follow-up during the first year of pre-antiretroviral therapy for HIV patients at sites providing pre-ART care in Nigeria, 2004–2012

**DOI:** 10.1371/journal.pone.0183823

**Published:** 2017-09-01

**Authors:** Simon G. Agolory, Andrew F. Auld, Solomon Odafe, Ray W. Shiraishi, E. Kainne Dokubo, Mahesh Swaminathan, Ibrahim Dalhatu, Dennis Onotu, Oseni Abiri, Henry Debem, Adebobola Bashorun, Tedd V. Ellerbrock

**Affiliations:** 1 Division of Global HIV & TB, U.S. Centers for Disease Control & Prevention, Atlanta, United States of America; 2 Division of Global HIV & TB, U.S. Centers for Disease Control & Prevention, Abuja, Nigeria; 3 School of Biomedical Informatics, University of Texas, Houston, United States of America; 4 National AIDS & STIs Control Program, Federal Ministry of Health, Abuja, Nigeria; University of Ottawa, CANADA

## Abstract

**Background:**

With about 3.4 million HIV-infected persons, Nigeria has the second highest number of people living with HIV (PLHIV) in the world. However, antiretroviral treatment (ART) coverage in Nigeria remains low with only 748,846 (22%) of PLHIV on ART by the end of 2014. Retention of HIV-infected patients in pre-ART care is essential to ensure timely ART initiation. We assessed outcomes of patients enrolled in Nigeria’s pre-ART program during 2004–2012.

**Methods:**

We conducted a nationally representative retrospective cohort study among adults (≥15 years old), enrolling in pre-ART programs supported by the U.S. President’s Emergency Plan for AIDS Relief in Nigeria. A total of 35 sites enrolling ≥50 patients in pre-ART were selected using probability proportional-to-size sampling; 2,415 eligible medical records at these sites were randomly selected for abstraction. Determinants of loss to follow-up (LTFU) and mortality during pre-ART care were estimated using Cox proportional hazards regression models.

**Results:**

The median age at enrollment was 32 years (interquartile range (IQR) 27–40). A total of 1,216 (51.4%) initiated ART by the time of data abstraction. Among the remaining 1,199 patients, 898 (74.9%) had been LTFU, 180 (15.0%) were alive and in pre-ART care, 71 (5.9%) had died, 50 (4.2%) had transferred out or stopped care. Baseline markers of advanced disease, including weight <45 kg (adjusted hazard ration (AHR) = 4.23; 95% confidence interval (CI): 1.51–15.58) and more advanced WHO disease stage, were predictive of pre-ART mortality. Compared with patients aged 15–24, patients aged 35–44 (AHR = 0.67; 95% CI: 1.0.47–0.95) and age 45–54 (AHR = 0.66; 95% CI: 0.48–0.91) had lower LTFU rates. Compared with attending facilities in North Central geopolitical zone, attending facility locations in South East (AHR = 0.44; 95% CI: 0.24–0.83) was protective against LTFU.

**Conclusions:**

About half of patients enrolling in HIV program during 2004–2012 in Nigeria had not initiated ART by 2013. Key strategies to improve early ART initiation among pre-ART enrollees include implementation of the WHO test and treat guidelines, earlier HIV testing, and better monitoring to improve ART initiation rates. Further research to understand regional variations in pre-ART outcomes is warranted.

## Introduction

Despite the changes in the antiretroviral therapy (ART) eligibility criteria over the last several years, which allow patients to be initiated on ART at relatively higher CD4 counts[1–6], the majority of patients in resource-limited settings who are starting ART continue to do so at lower CD4 counts, which is associated with higher morbidity and mortality[7–10]. There are many factors that account for late ART initiation, including late diagnosis of HIV, poor linkages to HIV care, delayed assessment for ART eligibility through CD4 testing or clinical staging, delayed initiation of ART for patients who are eligible, missed opportunities to identify ART-eligible patients through ongoing monitoring of patients found initially ineligible for ART, and poor retention of patients in pre-ART care [10–14].

Several studies from resource-limited settings have reported high rates of loss to follow-up (LTFU) and death after HIV diagnosis and before ART initiation [7, 11–14]. In a systematic review of retention in pre-ART care in sub-Saharan Africa, Plazy and colleagues found that up to 76% of patients who are not eligible for ART based on pre-ART CD4 testing at the HIV clinic are LTFU within the next 12 months[14].

Nigeria, with the second largest number of people living with HIV in the world, has one of the lowest ART coverage rates in the region; less than 25% (748,846) of the 3.4 million people living with HIV (PLHIV) in Nigeria were on ART by the end of 2014[15]. While limited HIV testing and poor linkage to treatment for those diagnosed HIV-positive contribute to low ART coverage, it is also likely that patients enrolled in care, who meet treatment eligibility, are not being initiated on ART[16]. We assessed the outcomes of patients enrolled in care at all sites supported by the U.S. President’s Emergency Plan for AIDS Relief (PEPFAR) throughout Nigeria during 2004–2012. PEPFAR supported the majority of HIV care and treatment sites in Nigeria during the evaluation period.

## Methods

We performed a retrospective study among a nationally representative sample of adults enrolled in HIV care in Nigeria. All PEPFAR-supported HIV facilities that had enrolled more than 50 HIV-infected adult patients (≥15 years old) in pre-ART care by Dec 31, 2012 were eligible for inclusion in the evaluation. Of these eligible facilities, 35 were selected using systematic probability proportional to size sampling (PPS), where size was based on the number of patients ever enrolled in pre-ART by Dec 31, 2012. Sites were implicitly stratified based on their location in the six geopolitical zones of Nigeria (North Central, North East, North West, South East, South-South, and South West) and size (small 51–150, medium 151–500, and large ≥500). At each of the selected 35 facilities, all adults, regardless of outcome at the time of chart abstraction, aged 15 years or older at the time of enrollment in care, who were enrolled during 2004–2012 and at least 12 months prior to the date of chart abstraction, were eligible for inclusion. We calculated the sample size required to achieve 95% confidence intervals of +2.5% and +3.0% around an estimated 12-month retention percentage of 75% for adult pre-ART patients. These calculations showed that a sample of 2,400 medical records from pre-ART sites would achieve the desired 95% confidence intervals, assuming a design effect of 3.0. Based on these calculations the planned sample size was 2,400 for pre-ART. We selected 69 charts per facility to get as close to our target sample size of 2,400 as possible.

HIV care and treatment guidelines in place in Nigeria during the study period recommended a comprehensive medical history, physical examination, CD4+ T-cell (CD4) count, and measurements of other bioclinical markers during the pre-ART care enrollment visit. For subsequent visits, the frequency of clinical assessments and testing for CD4 cells for pre-ART patients varied depending on baseline CD4 count and evolved over time. Before 2009, clinical assessments and testing for CD4 cells was recommended once every three months for patients with CD4 count below 500 cells/μL and twice a year for those with CD4 cells above 500 cells/μL. After 2009, clinical assessments were recommended every three months regardless of baseline CD4 count, while the recommendation for frequency of CD4 count testing remained the same.

During the evaluation period, ART eligibility criteria evolved following changes in World Health Organization (WHO) recommendations^1–7^ and was based on CD4 count levels and WHO clinical staging as follows: initially CD4 count ≤200 cells/μL or clinical stage 4 irrespective of CD4 cell count; in January 2007, the eligibility criteria changed to CD4 count ≤200 cells/μL or clinical stage 3/4 irrespective of CD4 count; and since March 2010, the criteria changed to CD4 count ≤350 cells/μL or WHO clinical stage 3/4.

In this evaluation, any patient not documented to have attended the HIV facility at least once during the 180 days prior to the date of data abstraction was counted as LTFU, unless the patient was known to have died, transferred-out to another facility, or known to have stopped care.

### Statistical analysis

Follow-up for the study participants started at the date of enrollment in pre-ART and ended at the earliest of death, transfer-out, ART initiation, or the date of last clinical visit. Patient characteristics were summarized over the complete study population. The number and percentage of missing data were tabulated. Percentages for categories were calculated using the number of patients with available data.

The proportion of patients by outcome (dead before ART start, started ART, still in pre-ART care, transferred out before ART start, stopped pre-ART, LTFU before ART start) were estimated. To estimate determinants of LTFU and mortality pre-ART, competing risk regression model by Fine and Gray[17] was used to estimate unadjusted and adjusted hazard ratios (AHRs), 95% confidence intervals (CI), and p-values for covariates of interest. The outcomes of death, LTFU and started on ART were considered competing risks because a patient who died could not subsequently become LTFU, or started on ART and a person observed to be LTFU in the database, could not subsequently be observed to have died or started on ART. We used the svyset and svy procedures to control for the complex survey design (i.e. clustering and weighting) of the study. Multiple imputations by chained equations were used to impute missing baseline clinical, and demographic data. The mi procedure in STATA was used to create 20 imputed datasets for each outcome and missing covariate data were imputed if <35% of data were missing. An adjusted sandwich variance estimator was used to adjust for clustering in the model. We used the stptime function in Stata[18] to estimate person-time and incidence rates per 100py for attrition, mortality, and LTFU over time in pre-ART care. All analyses were performed with STATA 13 (StataCorp. 2013. *Stata Statistical Software*: *Release 13*. College Station, TX: StataCorp LP) and SAS 9.3. (SAS Institute, Cary NC).

### Ethical approval and consent

Ethical approvals were obtained from the U.S. Centers for Disease Control and Prevention institutional review board (IRB) and the National Health Research Ethics Committee of Nigeria. Informed consent was not required due to the retrospective nature of this study and because no personally identifying information was collected.

## Results

### Characteristics of the study participants

A total of 2,415 patients were included in this evaluation; the median age at enrollment was 32 (interquartile range (IQR) 27–40) and 67.9% of participants were females of whom 11.3% were pregnant at time of enrollment. At enrollment, 60.7% of the study population were married, 19.3% had no education, and 57.9% were employed. Over one-third had undocumented or unknown HIV status of their partner/spouse. About 65% were referred to care and treatment services from voluntary counseling and testing (VCT) sites and <5% referred from antenatal (ANC) clinics and/or tuberculosis (TB) clinics. About 65% were at WHO clinical stage I or II and about 66% had CD4 count of ≤350 cells/ μL (Table 1).

**Table 1:** Sociodemographic and clinical characteristics of patients at pre-ART enrollment.

	Original Data			Following Multiple,Imputation (N = 2,415)
Patient characteristics	Un-weighted Frequency of Observation	Un-weighted Total	Weighted Percentages with (95% CI) OR Median with (IQR)	Weighted Median with IQR*, OR Percentages with (95% CI)
Sex^Ϯ^ No., N, %, (95% CI)				
Females	1640	2,415	67.9% (63.8–68.9)	67.9% (63.8–68.9)
Males	775	2,415	32.1% (31.2–36.2)	32.1% (31.2–36.2)
Age, No., N, %, (95% CI)				
15–24 years	367	2,415	15.2% (12.8–18.0)	14.5% (12.1–17.0)
25–34 years	1,071	2,415	44.4% (41.8–46.9)	44.7% (41.3–48.2)
35–44 years	627	2,415	26.0% (24.0–28.0)	26.0% (23.4–28.6)
45–54 years	258	2,415	10.7% (9.2–12.3)	10.8% (9.4–12.1)
>55 years	92	2,415	3.8% (3.2–4.5)	4.0% (3.4–4.5)
Median Age (years) No., N, median (IQR)				
Both sexes	2,415	2,415	32 (27–40)	32 (27–40)
Females	1,640	1,640	30 (25–37)	30 (25–37)
Males	775	775	37 (31–44)	37 (31–44)
Marital No., N, %, (95% CI)				
Single	418	2,271	18.4% (15.3–22.0)	20.1% (14.6–25.7)
Married	1,451	2,271	63.9% (60.1–67.6)	60.7% (56.5–64.9)
Divorced/Widowed/Others/	402	2,271	17.7% (15.3–20.4)	19.2% (15.1–23.2)
observations missing data**	144	2,415	6.0%	
Employment status No., N, %, (95% CI)				
Not employed	891	2,115	42.1% (34.3–50.4)	42.1% (30.7–53.5)
Employed	1,224	2,115	57.9% (49.6–65.7)	57.9% (46.5–69.3)
observations missing data**	300	2,415	12.4%	
Educational Status No., N, %, (95% CI)				
Tertiary	363	1,920	18.9% (15.5–22.9)	17.9% (14.5–21.2)
Secondary	583	1,920	30.5% (26.0–35.4)	33.2% (28.4–37.9)
Primary	556	1,920	29.0% (24.7–33.6)	29.7% (26.2–33.1)
No education	416	1,920	21.7% (15.2–30.9)	19.3% (13.4–25.2)
observations missing data**	495	2,415	20.5%	
Clinic size^Ϯ^ No., N, %, (95% CI)				
Large (≥1,500)	1,725	2,415	71.4% (53.6–84.4)	71.4% (53.6–84.4)
Medium (500–1499)	483	2,415	20.0% (9.5–37.4)	20.0% (9.5–37.4)
Small (<500 patients)	207	2,415	8.6% (2.6–24.6)	8.6% (2.6–24.6)
Region^Ϯ^				
North Central	828	2,415	34.3% (20.0–52.1)	34.3% (20.0–52.1)
North East	345	2,415	14.3% (5.8–31.1)	14.3% (5.8–31.1)
North West	414	2,415	17.1% (7.5–34.3)	17.1% (7.5–34.3)
South East	207	2,415	8.6% (2.6–24.6)	8.6% (2.6–24.6)
South-South	276	2,415	11.4% (4.1–27.8)	11.4% (4.1–27.8)
South West	345	2,415	14.3% (5.8–31.1)	14.3% (5.8–31.1)
Baseline Cd4 count, No., N, %, (95% CI)				
< = 100 cells/mm^3^	253	1094	23.1% (18.8–28.1)	23.8% (20.0–27.6)
101–200 cells/mm^3^	212	1094	19.4% (15.5–24.0)	19.3% (16.1–22.6)
201–350 cells/mm^3^	244	1094	22.3% (19.4–25.6)	23.3% (20.3–26.3)
>350 cells/mm^3^	385	1094	35.2% (27.3–44.1)	33.6% (28.6–38.6)
observations missing data**	1,321	2,415	54.7%	
Baseline Median CD4 Count, No., N, median (IQR)				
Both sexes	1,094	1,094	249 (102–424)	244 (106–428)
Females	750	1,094	259 (113–449)	260 (118–453)
Males	344	1,094	224 (76–375)	207 (90–384)
WHO Clinical Stage No., N, %, (95% CI)				
Stage I	845	1,648	39.2% (31.5–47.5)	37.8% (30.5–45.2)
Stage II	850	1,648	26.9% (22.4–32.0)	27.1% (22.1–32.2)
Stage III	1,323	1,648	27.9% (21.5–35.3)	30.2% (22.5–38.0)
Stage IV	246	1,648	6.1% (3.6–9.9)	4.8% (2.5–7.1)
observations missing data**	767	2,415	31.8%	
Partner/spouse HIV status				
HIV positive	375	1,408	26.6% (20.3–34.2)	23.8% (17.3–30.4)
HIV negative	320	1,408	22.7% (15.3–32.4)	23.4 (14.3–32.5)
Unknown	397	1,408	28.2% (18.0–41.2)	33.7% (22.1–45.2)
Does not have partner	316	1,408	22.4 (16.3–30.1)	19.1% (12.9–25.5)
observations missing data**	1,007	2,415	41.7%	
Care entry point				
VCT	1,564	2,329	67.2% (57.1–75.9)	64.5% (51.9–77.0)
PMTCT	92	2,329	4.0% (2.7–5.8)	3.6% (1.7–5.5)
Medical Outpatient	312	2,329	13.4% (8.2–21.1)	15.3% (7.5–23.0)
Others	361	2,329	15.5% (10.5–22.4)	16.7% (10.4–22.9)
observations missing data**	86	2,415	3.6%	
Baseline hemoglobin category No., N, %, (95% CI)				
Not Anemic	181	733	21.9% (18–26.3)	23.5% (17.8–29.2)
Mild Anemia	177	733	40.4% (37.7–43.0)	20.5% (16.9–24.2)
Moderate anemia	272	733	25.7% (22.7–29.0)	39.7% (35.2–44.3)
Severe anemia	103	733	12.1% (9.6–15.0)	16.2% (10.1–22.3)
observations missing data**	1,682	2,415	69.6%	
Baseline Median hemoglobin No., N, % median (IQR)				
Both sexes	733	733	10.8 (9–12)	10.7 (9.0–12.26)
Females	504	733	10.6 (9–11.8)	10.5 (8.9–12.0)
Males	229	733	11.0 (9.4–13.0)	11.12 (9.4–12.9)
Baseline weight category No., N, %, (95% CI)				
60 kg	713	2,017	35.2% (31.2–39.3)	36.4% (31.8–41)
45–60 kg	1,014	2,017	52.4% (49.4–55.5)	50.7% (47.5–54)
<45kg	290	2,017	12.5% (10.5–14.8)	12.9% (10.2–15.5)
observations missing data**	398	2,415	16.5%	
Baseline Median weight No., N, % median (IQR)				
Both sexes	2,017	2,017	56.0 (48–65)	56.0 (48–65)
Females	1,373	2,017	54.0 (46–63)	54.6 (46.1–63.5)
Males	644	2,017	60.0 (53–68)	60 (54–68)
CTX Status				
No	1,719	2,237	76.8% (67.6–84.1)	80.1% (72.3–87.8)
Yes	518	2,237	23.2% (15.9–32.4)	19.9% (12.2–27.7)
observations missing data**	178	2,415	7.4%	
Year of ART start^Ϯ^ No., N, %, (95% CI)				
2004–2006	248	2,415	10.3% (6.5–15.9)	10.7% (4.5–17.0)
2007–2009	1,283	2,415	53.2% (48.7–57.6)	55.3% (51.9–58.8)
2010–2012	882	2,415	36.6% (30.2–43.5)	33.9% (28.0–39.8)

CTX Status: Patient receiving co-trimoxazole. Abbreviations: CI, confidence interval; IQR, interquartile range; WHO, World Health Organization; Kgs, kilograms; ART, antiretroviral therapy; D4T, stavudine; 3TC, lamivudine; NVP, nevirapine; EFV, efavirenz; AZT, zidovudine; ABC, abacavir; CTX, co-trimoxazole. Classification of anemia in males: Mild = Hemoglobin Concentration (HBC) 11–12.9g/dl; Moderate: HBC 8–10.9g/dl and severe: HBC 10.9 - <8g/dl (WHO classification). Classification of anemia in females: Mild = (HBC) 11–11.9g/dl; Moderate: HBC 8–10.9g/dl and severe: HBC <8g/dl (WHO classification)

*Median and IQR calculated across 60 imputed datasets.

ϮVariables with complete data.

**Unweighted sample estimate.

### Rates of ART initiation and attrition among pre-ART patients

A total of 1,216 (51.4%) initiated ART by the time of data abstraction. Among the remaining 1,199 patients, 898 (74.9%) had been LTFU, 180 (15.0%) were alive and in pre-ART care, 71 (5.9%) had died during pre-ART care, 50 (4.2%) had transferred out or stopped care.

During 1677.99 person-years (py) of follow up, the overall incidence of attrition was 62.97/100 py which included (59.51/100 py) incidence of LTFU and (3.46/100py) incidence of mortality. The incidence rate of ART initiation was 70.43/100 py (Table 2).

**Table 2:** Rates of attrition, mortality, and LTFU from pre-ART service.

Duration in care (years)	person-years (py)	Attrition rate/100py	Mortality rate/100py	LTFU (rate/100py)	ART initiation (rate/100py)
**0-.25**	312.42	220.6	8.17	212.43	245.33
**.25-.5**	205.14	40.97	5.22	35.75	54.72
**.5–1**	313.38	25.8	2.51	23.29	33.89
**1–2**	396.46	29.52	2.08	27.44	23.02
**2–3**	215.46	22.49	1.14	21.35	21.2
**3–4**	117.88	15.8	1.8	14	22.08
**4–5**	60.35	5.25	1.81	3.44	25.66
**5–6**	33.97	28.57	0	28.57	19.3
**6–7**	16.96	6.9	0	6.9	43.9
**>7**	5.98	73.09	0	73.09	74.45
**Total**	1677.99	62.97	3.46	59.51	70.43

At 6, 12 and 24 months similar percentages of eligible males and females were started on ART; 37% vs. 36%, 41% vs. 41%, and 44% vs. 43%, respectively. Many patients eligible for treatment using CD4 count criteria were not started on ART and were still in HIV care; 19% at 6 months, 12% at 12 months and 9% at 24 months.

### Patients’ characteristics associated with mortality and lost to follow up

During pre-ART care, baseline markers of advanced disease, including weight <45 kg compared to weight >60kg (AHR = 4.23; 95% CI: 1.51–15.58), and more advanced WHO disease stages compared to stage I; stage III (AHR = 3.99; 95% CI: 1.08–14.76) and stage IV (AHR = 12.67; 95% CI: 3.00–53.40), were predictive of pre-ART mortality. In addition, compared to being unemployed, being employed was also associated with pre-ART mortality (AHR = 2.38; 95% CI 1.11–5.10). Table 3 shows other baseline patient characteristics and their association with mortality during pre-ART care.

**Table 3:** Patient characteristics at pre-ART enrolment associated with mortality.

Variable	Unadjusted Haz. Ratio (95%CI)	p value	Adjusted Haz. Ratio (95%CI)	p value
**Age**				
**15–24**	1.0		1.0	
**25–34**	1.42 (0.56–3.57)	0.46	1.51 (0.63–3.64)	0.36
**35–44**	1.88 (0.61–5.82)	0.27	1.73 (0.60–4.98)	0.30
**45–54**	1.69 (0.72–3.96)	0.23	1.69 (0.59–4.83)	0.33
**>55**	1.37 (0.43–4.36)	0.60	0.85 (0.14–5.27)	0.86
**Weight**				
**>60 kg**	1.0		1.0	
**45–60 kg**	2.37 (0.81–6.95)	0.12	1.55 (0.54–4.49)	0.42
**<45 kg**	8.22 (3.14–21.50)	0.00	4.23 (1.51–15.58)	0.02
**Hemoglobin**				
**Not anemic**	1.0		1.0	
**Mild Anemia**	1.67 (0.52–5.41)	0.39	1.07 (0.32–3.60)	0.91
**Moderate Anemia**	1.54 (0.45–5.29)	0.49	0.55 (0.14–2.13)	0.43
**Severe Anemia**	2.18 (0.40–11.91)	0.37	0.35 (0.05–2.72)	0.43
**CD4 cell count**				
**≤100**	1.0		1.0	
**101–200**	0.82 (0.36–1.85)	0.63	1.23 (0.46–2.92)	0.65
**201–350**	0.55 (0.18–1.61)	0.27	1.08 (0.33–3.42)	0.90
**>350**	0.17 (0.05–0.58)	0.01	0.40 (0.13–3.03)	0.56
**Sex**				
**Female**	1.0		1.0	
**Male**	1.59 (1.0–2.53)	0.05	1.1 (0.57–2.12)	0.78
**Employment status**				
**Not employed**	1.0		1.0	
**Employed**	1.71 (0.74–3.95)	0.21	2.38 (1.11–5.10)	0.03
**Educational Status**				
**Tertiary**	1.0		1.0	
**Secondary**	0.86 (0.36–2.07)	0.74	0.78 (0.23–2.62)	0.70
**Primary**	0.76 (0.36–1.59)	0.46	0.78 (0.27–2.17)	0.52
**None**	1.73 (0.45–6.60)	0.42	1.19 (0.45–3.13)	0.73
**clinic Size**				
**Large (≥1,500)**				
**Medium (500–1,499)**	2.89 (0.79–10.50)	0.11	1.53 (0.72–3.98)	0.37
**Small (<500 patients)**	1.45 (0.32–6.60)	0.63	1.58 (0.1–26.81)	0.75
**Geographical Region**				
**North Central**	1.0		1.0	
**North East**	5.40 (0.60–50.0)	0.13	3.57 (0.36–34.99)	0.27
**North West**	1.57 (0.43–5.76)	0.49	1.12 (0.10–16.77)	0.93
**South East**	0.44 (0.05–3.62)	0.45	0.47 (0.10–4.08)	0.50
**South South**	4.37 (1.14–16.71)	0.03	3.76 (0.93–15.24)	0.06
**South West**	0.91 (0.09–9.33)	0.94	1.17 (0.11–12.88)	0.90
**Marital Status**				
**Single**	1.0		1.0	
**Married**	0.49 (0.31–0.77)	0.00	0.49 (0.21–1.07)	0.07
**Divorce/widowed/others**	0.64 (0.33–1.21)	0.17	0.50 (0.21–1.20)	0.12
**CTX Status**				
**No**	1.0		1.0	
**Yes**	3.41 (1.95–5.95)	0.00	1.71 (0.83–3.50)	0.23
**Year of enrolment in Care**				
**2004–2006**	1.0		1.0	
**2007–2009**	0.97 (0.34–2.78)	3.33	0.46 (0.15–1.45)	0.19
**2010–2012**	1.68 (0.60–4.72)	7.61	0.89 (0.35–2.72)	0.85
**WHO Stage**				
**Stage I**	1.0		1.0	
**Sage II**	3.51 (1.01–12.20)	0.05	2.30 (0.63–8.35)	0.21
**Stage III**	7.84 (2.31–26.59)	0.00	3.99 (1.08–14.76)	0.04
**Stage IV**	25.92 (7.35–91.37)	0.00	12.67 (3.00–53.40)	0.001

Compared with patients aged 15–24, patients aged 35–44 (AHR = 0.67; 95% CI: 0.47–0.95) and 45–54 (AHR = 0.66; 95% CI: 0.48–0.91) had lower LTFU rates. Compared with attending facilities in the North Central geopolitical zone, attending facility locations in the South East (AHR = 0.44; 95% CI: 0.24–0.83) was protective against LTFU. Predictors of LTFU included weight <45 kg (AHR = 1.45; 95% CI: 1.06–1.99) and weight 45–60 kg (AHR = 1.23; 95% CI: 1.03–1.49) compared to weight ≥60kg; moderate (AHR = 1.66; 95% CI: 1.15–2.39) and severe anemia (AHR = 2.16; 95% CI: 1.37–3.38), and male gender (AHR = 1.47; 95% CI: 1.14–1.75). Table 4 displays other baseline patient characteristics associated with LTFU during pre-ART care.

**Table 4:** Patient characteristics at Pre-ART enrolment associated with LTFU.

Variables	Un-Adjusted Haz. Ratio (95%CI)	p value	Adjusted Haz. Ratio (95%CI)	p value
**Age**				
**15–24**	1.0		1.0	
**25–34**	0.82 (0.64–1.04)	0.11	0.86 (0.67–1.10)	0.22
**35–44**	0.71 (0.54–0.93)	0.01	0.67 (0.47–0.95)	0.02
**45–54**	0.73 (0.57–0.94)	0.01	0.66 (0.48–0.91)	0.01
**>55**	1.03 (0.64–1.64)	0.90	0.96 (0.62–1.48)	0.86
**Weight**				
**>60 kg**	1.0		1.0	
**45–60 kg**	1.38(1.13–1.69)	0.00	1.23 (1.03–1.49)	0.03
**<45 kg**	1.89 (1.45–2.46)	0.00	1.45 (1.06–1.99)	0.02
**Hemoglobin**				
**Not anemic**	1.0		1.0	
**Mild Anemia**	1.41 (1.03–1.93)	0.03	1.36 (0.98–1.89)	0.06
**Moderate Anemia**	1.69(1.21–2.36)	0.00	1.66 (1.15–2.39)	0.01
**Severe Anemia**	2.38 (1.60–3.54)	0.00	2.16 (1.37–3.38)	0.00
**CD4 Cell Count**				
**≤100**	1.0		1.0	
**101–200**	0.81 (0.55–1.20)	0.29	0.91 (0.62–1.33)	0.63
**201–350**	0.79 (0.53–1.16)	0.22	0.91 (0.62–1.35)	0.65
**>350**	0.76 (0.48–1.20)	0.24	1.06 (0.66–1.71)	0.80
**Sex**				
**Female**	1.0		1.0	
**Male**	1.2 (1.02–1.42)	0.02	1.47 (1.14–1.75)	0.00
**Employment Status**				
**Not employed**	1.0		1.0	
**Employed**	1.05 (0.83–1.34)	0.68	1.02 (0.84–1.24)	0.86
**Educational Status**				
**Tertiary**	1.0		1.0	
**Secondary**	1.36 (1.04–1.76)	0.02	1.29 (0.95–1.75)	0.10
**Primary**	1.29 (1.05–1.60)	0.02	1.22 (0.96–1.55)	0.10
**None**	1.29 (0.99–1.69)	0.06	1.44 (0.97–2.14)	0.07
**Clinic Size**				
**Large (≥1,500)**	1.0		1.0	
**Medium (500–1,499)**	0.92 (0.45–1.87)	0.81	0.84 (0.58–1.23)	0.36
**Small (<500 patients)**	0.72 (0.41–1.25)	0.24	0.84 (0.58–1.19)	0.32
**Geographical region**				
**North Central**	1.0		1.0	
**North East**	0.52 (0.29–0.92)	0.03	0.53 (0.24–1.14)	0.10
**North West**	0.93 (0.63–1.37)	0.72	0.73 (0.42–1.29)	0.28
**South East**	0.39 (0.19–0.78)	0.01	0.44 (0.24–0.83)	0.01
**South South**	1.37 (0.90–2.07)	0.14	1.57 (0.95–2.60)	0.08
**South West**	1.16 (0.69–1.94)	0.58	1.70 (0.92–3.15)	0.09
**Marital Status**				
**Single**	1.0		1.0	
**Married**	0.86 (0.73–1.01)	0.07	0.96 (0.80–1.16)	0.68
**Divorce/widowed/others**	0.84 (0.70–1.01)	0.06	1.02 (0.82–1.28)	0.85
**CTX Status**				
**No**	1.0		1.0	
**Yes**	1.15 (0.83–1.60)	0.39	1.01 (0.69–1.49)	0.95
**Year of enrolment in care**				
**2004–2006**	1.0		1.0	
**2007–2009**	1.39 (0.98–1.96)	0.06	1.11 (0.76–1.65)	0.58
**2010–2012**	1.45 (0.91–2.31)	0.12	0.99 (0.65–1.51)	0.97
**WHO Stage**				
**Stage I**	1.0		1.0	
**Sage II**	0.76 (0.55–1.03)	0.08	0.67 (0.48–0.92)	0.01
**Stage III**	1.30 (0.97–1.74)	0.08	0.98 (0.71–1.37)	0.34
**Stage IV**	1.31 (0.76–2.23)	0.34	0.89 (0.5–1.57)	0.42

## Discussion

Enrollment and retention of patients in HIV care are critical steps in providing patients with appropriate services to reduce morbidity and mortality from HIV. Nigeria, with the second highest number of PLHIV globally, continues to face challenges in enrolling patients on ART. This study, one of the first in Nigeria to evaluate outcomes among patients once enrolled in pre-ART care, paints a stark picture—nearly half of patients who were enrolled in care during 2004–2012 had not been initiated on ART by the end of 2012. This suggests that the majority of patients in our study continued to be at high risk of morbidity and mortality from HIV and HIV-associated diseases despite knowing their HIV-positive status and having been linked to care.

Many factors may be contributing to none or delayed ART initiation among patients who are enrolled. A significant proportion of patients are LTFU before initiating ART. Patients who are younger or older at baseline and male patients were found to be significantly at increased risk of LTFU. This is consistent with findings from other studies from Sub-Saharan Africa [19]. Having services that are tailored to unique needs of these populations, e.g., teen clubs, and decentralizing to provide care that is closer to patient homes can help reduce LTFU [9, 20–22]. Our study also demonstrated that patients from South East geopolitical zone compared to patients from North Central, where significantly at lower risk of LTFU. It is unclear what the underlying reasons are but the North Central geopolitical zone, especially the city of Jos in Plateau State and the surrounding areas, prior to and during the time of the evaluation was affected by communal strife and many patients could have been displaced to other areas of Nigeria [23]. National and regional HIV programs should develop appropriate contingency plans for PLHIV in conflict affected areas of Nigeria to ensure continuity of services during displacement [24, 25]. Implementation of better patient tracking systems and use of innovative systems like case reporting and unique patient identification number can help mitigate some issues surrounding undocumented transfers [26].

The clinical characteristics found to be associated with LTFU in our study, which are similar to those reported elsewhere, include being under weight, having moderate to severe anemia, and having WHO stages III and IV at baseline [7, 9–13]. Early HIV diagnosis and prompt linkages to care can help reduce the number of patients who are enrolling in care with advanced HIV disease. A significant number of our patients had partners with unknown HIV status. Several studies from sub-Saharan Africa have shown that partners and children of HIV-infected patients who are already enrolled in care have higher rates of HIV positivity when tested compared to the general population [27–33]. Effort should be exerted to ensure that partners and family members of HIV-infected patients are prioritized for HIV testing. Most of our patients were referred to pre-ART services from VCT centers and less than 5% were referred from TB and STI clinics. Patients with active TB disease and patients with STIs continue to be diagnosed with HIV at a higher rate compared to general populations [27, 28, 34]. HIV testing and counseling services should be strengthened at TB and STI clinics to ensure that patients seen at these clinics are tested for HIV and those found to be HIV-positive are promptly referred to HIV care and treatment services.

The rates and predicators of mortality among our patients were similar to what is reported from other ART programs in Africa [7, 9–13]. Patients enrolling in pre-ART program with advanced HIV disease including patients with stage III and IV disease and low weight were found to be at increased risk for mortality. Early HIV diagnoses and ART initiation among patients enrolling in care can help reduce mortality [35, 36]. Health information systems which alert healthcare providers to check ART eligibility for patients not on ART may be helpful in ensuring patients get the appropriate clinical evaluations on time and get initiated on ART when they meet national eligibility criteria [37]. Adopting the new WHO test and treat recommendations can help streamline the process of evaluating patients for ART eligibility and allow healthcare providers to initiate patients on ART early [38]. Differential models of care, including community-based ART programs and fewer clinic appointments for stable patients [20, 21], can help decongest many ART sites and allow healthcare workers to focus on meeting the needs of patients who need additional support.

Our study also demonstrated an association between being employed and mortality. It is unclear why being employed is a risk factor for mortality. Studies from developed countries have shown negative association between being employed and mortality, referred to as the “healthy worker effect”[39]. However, in most developed countries there are protections for workers, such as sick leave that allow workers to miss work when sick to access health services. Many ART clinics in Africa, especially the large facilities, tend to have long wait times for patients [20]. Patients who are employed, especially as manual laborers, because of economic insecurities, may not be able to take leave from work for several hours a day every 1–3 months to access needed services [40]. Interventions, such as educating workers on their rights and employers on the benefits of a healthy workforce, and strengthening workers protections may help improve access to healthcare and outcomes among employed HIV-infected patients [41].

There are some limitations to our study. We were not able to collect data on socio-economic factors from study participants since we abstracted our data from patient charts and facility registers and these sources did not contain this information. This limited our ability to look at other factors that may be associated with outcomes among pre-ART patients. Because of the retrospective nature of our study, there were some data fields and variables that were missing. For example, as in similar evaluations using routine data [8, 42], our estimates of patients LTFU most likely included patients who have died but deaths were not documented. This highlights the importance of developing newer and innovative monitoring and evaluation tools for better monitoring of quality of care provided to patients in HIV in care.

## Conclusion

About half of patients enrolling in the HIV program in Nigeria during 2004–2012 were not initiated on ART by the end of 2012. Implementation of the WHO test and treat guidelines, decentralization of HIV services, implementation of differentiated models of care, and better monitoring tools at current HIV sites may help improve ART initiation rates among patients enrolling in care in Nigeria. Additional studies are needed to understand how certain factors (e.g., being employed) impact outcomes among pre-ART patients.

## Disclaimer

The findings and conclusions in this manuscript are those of the authors and do not necessarily represent the views of the United States (U.S.) Centers for Disease Control and Prevention. Use of trade names is for identification only and does not imply endorsement by the U.S. Centers for Disease Control and Prevention or the U.S. Department of Health and Human Services.

## Supporting information

S1 File Minimal dataset. (DTA)
